# Adrenal ganglioneuroma in an adolescent presenting as an incidentaloma: Imaging–pathology correlation and diagnostic challenges: A case report

**DOI:** 10.1097/MD.0000000000051533

**Published:** 2026-11-06

**Authors:** Wasef Alhroub, Omair Bseiso, Mohammad Manasra, Muhammad Sweity, Mohammad Alami, Karam M Hmidat, Osaid Dabbas, Ameer Qawasmi, Aya Abedallah Ibrahim, Hani Rjoob, Ammar Hassouneh, Sami Bannoura, Abdallah Altell, Izzeddin A Bakri

**Affiliations:** a Faculty of Medicine, Hebron University, Hebron, Palestine; b Endocrinology and Metabolism, Faculty of Medicine, Palestine Polytechnic University, Hebron, Palestine; c Department of Radiology, Hebron Governmental Hospital, Hebron, Palestine; d Department of Pathology, Al-Makassed Hospital, Jerusalem, Palestine; e Department of Pathology, Faculty of Medicine, Al-Quds University, Jerusalem, Palestine.

**Keywords:** adolescent, adrenal ganglioneuroma, adrenal incidentaloma, case report, delayed enhancement, neurogenic tumor

## Abstract

**Rationale:**

Adrenal ganglioneuroma (AGN) is a rare, benign, and usually nonfunctioning tumor arising from neural crest-derived sympathetic ganglion cells. Preoperative diagnosis is difficult because the clinical presentation is often nonspecific, and imaging findings overlap with those of other adrenal neoplasms. We describe an adolescent adrenal incidentaloma with detailed imaging–pathology correlation, adding to the small number of reported adolescent cases.

**Patient concerns:**

A previously healthy 16-year-old male underwent contrast-enhanced computed tomography (CT) after a bicycle accident. No traumatic injury was identified, but imaging incidentally revealed a well-circumscribed right adrenal mass measuring approximately 6.1 cm × 3.6 cm × 5.0 cm.

**Diagnoses:**

CT and magnetic resonance imaging showed progressive, persistent enhancement across the arterial, venous, and delayed phases without demonstrable washout (attenuation increased from 20 Hounsfield units unenhanced to 55 Hounsfield units in the venous phase, with further qualitative enhancement on 15-minute delayed images) and no intralesional fat, suggesting a non-adenomatous lesion and raising suspicion for a neurogenic tumor. Biochemical evaluation showed no evidence of hormonal hypersecretion. Histopathological examination after resection established the diagnosis of AGN.

**Interventions:**

The patient underwent right adrenalectomy because of the size and radiologically indeterminate nature of the mass.

**Outcomes:**

The postoperative course was uneventful. Transient elevation of blood pressure noted during follow-up prompted biochemical reevaluation, which showed no evidence of excess catecholamine. Follow-up CT at 9 months showed no recurrence.

**Lessons:**

Progressive delayed enhancement without washout and absence of signal loss on opposed-phase magnetic resonance imaging, a pattern previously described in ganglioneuroma, may suggest the diagnosis preoperatively, although definitive diagnosis requires histopathological confirmation. Complete surgical excision established the diagnosis and achieved a favorable short-term outcome; the excellent long-term prognosis of AGN is supported by the published literature, and our patient remains recurrence-free at 9 months.

## 1. Introduction

Adrenal ganglioneuroma (AGN) is a rare benign tumor arising from sympathetic ganglion cells derived from the neural crest within the adrenal medulla. It represents the most mature entity within the neuroblastic tumor spectrum, which also includes ganglioneuroblastoma and neuroblastoma.^[1,2]^

AGN is uncommon among adrenal tumors. In a large adrenalectomy series, AGN accounted for only 35 of 1784 cases (1.9%), underscoring the rarity of this lesion.^[3]^ Most AGNs are hormonally inactive and asymptomatic, and they are frequently discovered incidentally on imaging performed for unrelated reasons.^[4,5]^

Radiologic evaluation typically demonstrates a well-defined adrenal mass, but imaging findings often overlap with those of adenoma, pheochromocytoma, adrenocortical carcinoma (ACC), and metastasis. Progressive delayed enhancement and the absence of intralesional fat may suggest a neurogenic lesion, although histopathology remains essential for definitive diagnosis.^[5-7]^

We report an incidentally detected right AGN in a 16-year-old male and describe the imaging–pathology correlation that guided diagnostic reasoning and definitive management. Rather than proposing a new imaging sign, this case adds to the small number of reported adolescent AGNs and illustrates how a previously described enhancement pattern, combined with structured biochemical evaluation, can narrow the differential diagnosis of an adrenal incidentaloma in a young patient. This report was prepared in accordance with the CARE (CAse REport) guidelines, and a timeline of the clinical course is provided in Table 1.

**Table 1:** Timeline of the clinical course.

Time point	Event
Day 0	Bicycle accident with handlebar trauma; emergency department evaluation; contrast-enhanced abdominal CT to exclude visceral injury – incidental right adrenal mass (6.1 cm × 3.6 cm × 5.0 cm) identified
Diagnostic work-up*	Multiphase adrenal-protocol CT including a 15-min delayed phase; biochemical evaluation (PRA, cortisol, aldosterone, plasma metanephrines, catecholamines; 24-h urinary VMA) – no hormonal hypersecretion; adrenal-protocol MRI – no chemical-shift signal loss, progressive enhancement without demonstrable washout
Surgical treatment*	Right adrenalectomy
Histopathology*	Adrenal ganglioneuroma – mature ganglion cells within Schwannian stroma; no immature neuroblastic elements after extensive sampling
Early postoperative follow-up*	Uneventful recovery; transient elevation of blood pressure noted; repeat 24-h urinary metanephrines, normetanephrines, VMA, and catecholamines – normal
9 mo postoperatively	Follow-up CT – no local recurrence, no new lesion

CT = computed tomography, MRI = magnetic resonance imaging, PRA = plasma renin activity, VMA = vanillylmandelic acid.

* Events are shown in verified chronological order; the exact calendar intervals between these intermediate steps could not be retrieved from the records and are therefore not shown.

## 2. Case presentation

In accordance with the policy of the Faculty of Medicine, Hebron University, ethics committee approval is not required for the reporting of individual anonymized cases; written informed consent for publication of anonymized clinical information and images was obtained from the patient’s legally authorized representative.

A 16-year-old male student, previously healthy, presented to the emergency department with generalized body pain after a bicycle accident (day 0; Table 1). He had lost control and struck the bicycle handlebars, sustaining direct trauma to the left lower chest, left hypochondrium, and epigastric region. He denied loss of consciousness, vomiting, or seizures.

On examination, the patient was alert, conscious, and hemodynamically stable, with a Glasgow Coma Scale score of 15/15. Physical findings were limited to bruising and tenderness over the left lower anterior chest, along with mild epigastric tenderness. The remainder of the systemic examination was unremarkable. Vital signs and routine laboratory investigations were within normal limits.

A contrast-enhanced abdominal computed tomography (CT) scan was obtained to exclude traumatic visceral injury. Although no traumatic sequelae were identified, the scan revealed an incidental, well-defined right adrenal solid mass measuring approximately 6.1 cm × 3.6 cm × 5.0 cm. On adrenal-protocol CT, the lesion measured 20 Hounsfield units (HU) on unenhanced images, increasing to 35 HU in the arterial phase and 55 HU in the portal venous phase; delayed 15-minute images demonstrated persistent, further increasing enhancement without visually appreciable washout (Fig. 1). The numeric delayed-phase attenuation value could not be retrieved from the archived imaging records for this revision; formal absolute and relative percentage washout values therefore could not be calculated, and the assessment of absent washout rests on the qualitative multiphase appearance in conjunction with the documented rise in attenuation from 20 to 55 HU (see the limitations discussed below). No macroscopic fat, calcification, adjacent invasion, or local extension was seen.

**Figure 1.**
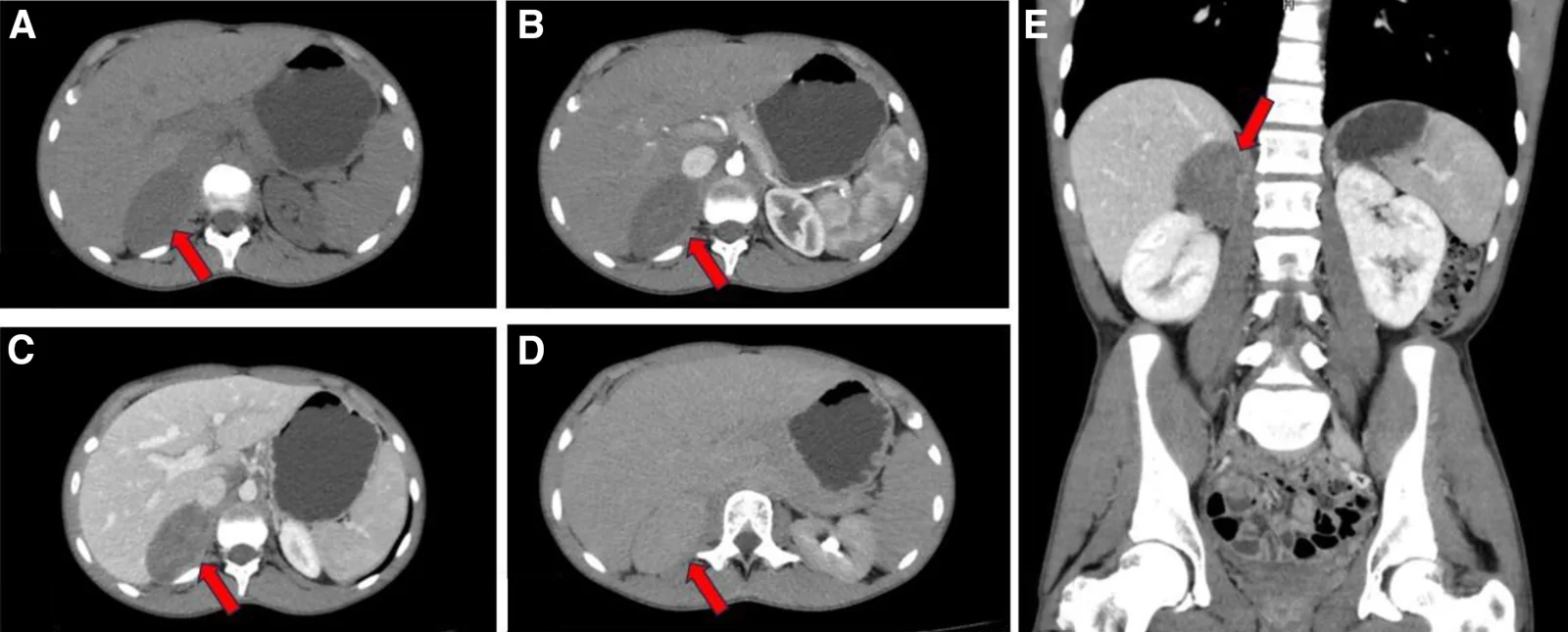
Axial contrast-enhanced CT adrenal protocol shown in multiple phases: (A) pre-contrast, (B) arterial, (C) venous, and (D) delayed 15-minute phase, demonstrating a well-defined right adrenal solid mass (red arrow) measuring approximately 6.1 cm × 3.6 cm, without fat density. The lesion shows gradual and persistent enhancement, most conspicuous on the delayed phase. No adjacent invasion is seen. (E) Coronal reformatted CT adrenal protocol image showing the right adrenal solid mass (red arrow) with smooth margins and no local invasion. CT = computed tomography.

Because the mass was discovered incidentally, biochemical evaluation was performed to assess hormonal function. Supine plasma renin activity was mildly elevated at 5.4 ng/mL/h. Serum cortisol, plasma aldosterone, plasma metanephrines, and catecholamines were within the laboratory reference intervals, and 24-hour urinary vanillylmandelic acid (VMA) excretion was 3.5 mg/d, within normal limits. The original laboratory reports, including the assay methodology and the institution-specific reference intervals, could not be retrieved for this revision; results are therefore reported as documented in the clinical record, with the laboratory’s classification of values as normal or elevated (see the limitations discussed below). Overall, there was no biochemical evidence of catecholamine excess, hypercortisolism, or primary aldosteronism, and the lesion was considered nonfunctioning.

Adrenal-protocol magnetic resonance imaging (MRI) further characterized the lesion as a well-circumscribed right adrenal mass. Chemical-shift imaging showed no signal drop on opposed-phase images, excluding significant intracellular lipid. Dynamic post-contrast T1-weighted images mirrored the CT findings, demonstrating progressive enhancement that peaked on delayed images without washout (Fig. 2). These features were atypical for a lipid-rich adrenal adenoma and raised the possibility of a neurogenic tumor.

**Figure 2.**
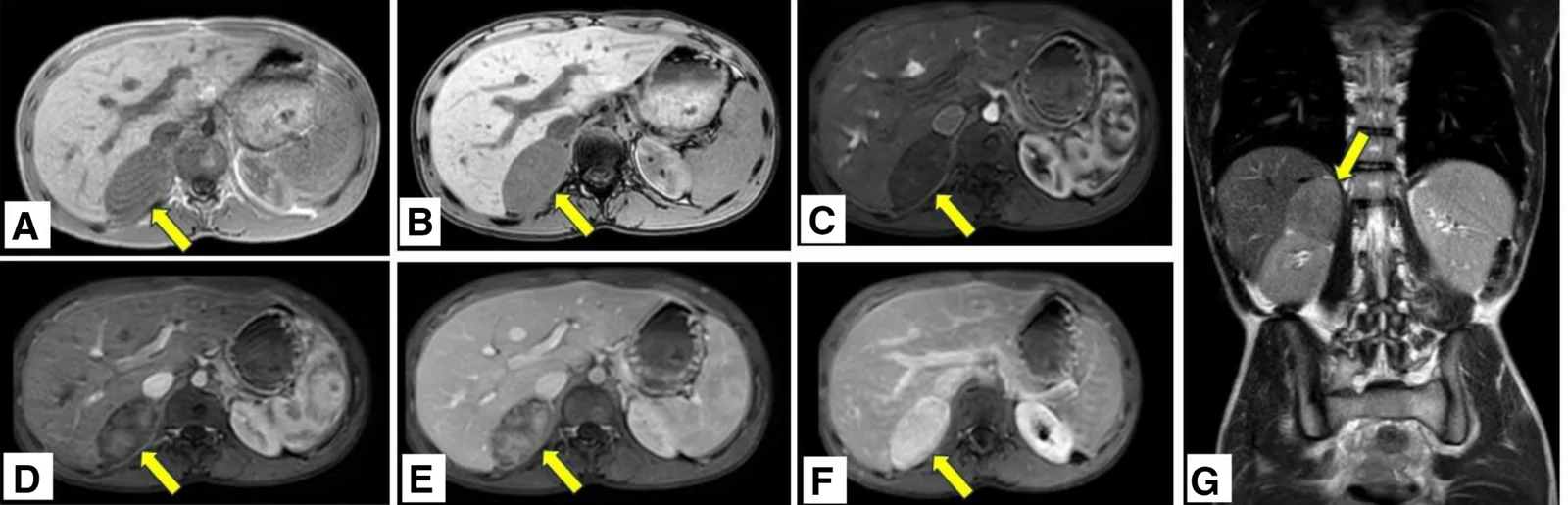
MRI adrenal protocol: (A) axial in-phase image demonstrating a well-defined right adrenal mass (yellow arrow); (B) opposed-phase image showing no signal drop within the lesion, excluding significant intralesional fat; (C–E) dynamic post-contrast T1-weighted axial images demonstrating progressive and persistent enhancement of the right adrenal mass (yellow arrow); (F) axial delayed post-contrast image showing the highest relative enhancement of the lesion; and (G) coronal T2-weighted image again showing the adrenal lesion without invasion of adjacent organs. MRI = magnetic resonance imaging.

The preoperative differential diagnosis included adrenal adenoma (considered unlikely given the unenhanced attenuation of 20 HU, the absence of chemical-shift signal loss, and the lack of washout), pheochromocytoma (biochemically excluded, although imaging overlap can occur), ACC, and, given the patient’s age, less mature neuroblastic tumors, namely ganglioneuroblastoma and neuroblastoma, which could not be excluded without tissue diagnosis. In view of the lesion’s size (>6 cm in maximal diameter), its radiologically indeterminate enhancement pattern, and the patient’s young age, the decision was made to proceed with definitive resection, and the patient underwent right adrenalectomy.

Gross examination showed a well-defined, oval mass measuring 7.0 cm × 5.5 cm × 3.8 cm and weighing 70 g, slightly larger than the radiologic estimate (see Discussion). The cut surface was tan and whorled, without hemorrhage or necrosis. Histopathological examination demonstrated a well-circumscribed tumor composed of spindle-shaped Schwann cells within a fibrous stroma and scattered mature ganglion cells, with no immature neuroblastic elements identified after extensive tissue sampling and no evidence of malignancy. These morphological findings were diagnostic of ganglioneuroma; immunohistochemistry showing S100 positivity in both the Schwannian and ganglion cell components provided supportive evidence of the neural origin of both tumor components (Fig. 3).

**Figure 3.**
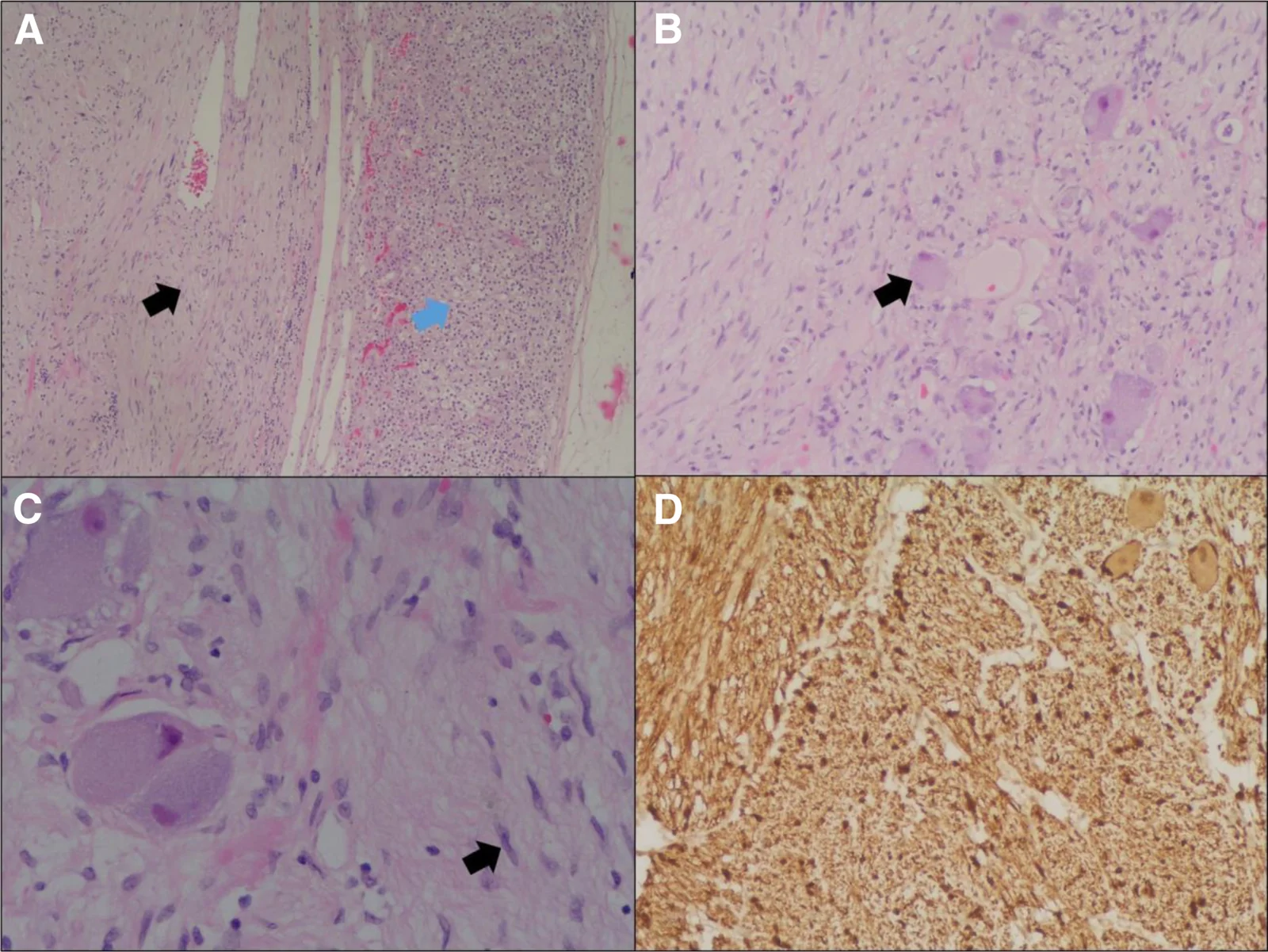
(A) Sections show a well-circumscribed adrenal medulla tumor (black arrow) with a sharp interface with the adrenal cortex (blue arrow; H&E, 4×). (B) The tumor is composed of Schwann cells/stroma and mature ganglion cells (H&E, 10×). (C) Spindle cells with elongated, bland, and wavy nuclei, characteristic of Schwann cells (arrow), are present; mature ganglion cells are seen on the left side of the field (H&E, 20×). (D) Both components of ganglioneuroma are positive for S100 immunostain (10×). No immature neuroblastic elements were seen after extensive tissue sampling. H&E = hematoxylin and eosin.

The immediate postoperative course was uneventful. During outpatient follow-up, elevated blood pressure readings were noted. Given the history of an adrenal tumor, biochemical reevaluation was performed to exclude a functional lesion or a missed pheochromocytoma: 24-hour urinary metanephrines, normetanephrines, VMA, and catecholamines were all within normal limits. The elevation was transient on clinical follow-up; however, because the individual measurements, their exact timing, and their duration were not systematically documented and could not be retrieved for this revision, no conclusion is drawn regarding its cause (see the limitations discussed below). Follow-up CT performed 9 months after adrenalectomy showed no local recurrence and no new lesion (Fig. 4).

**Figure 4.**
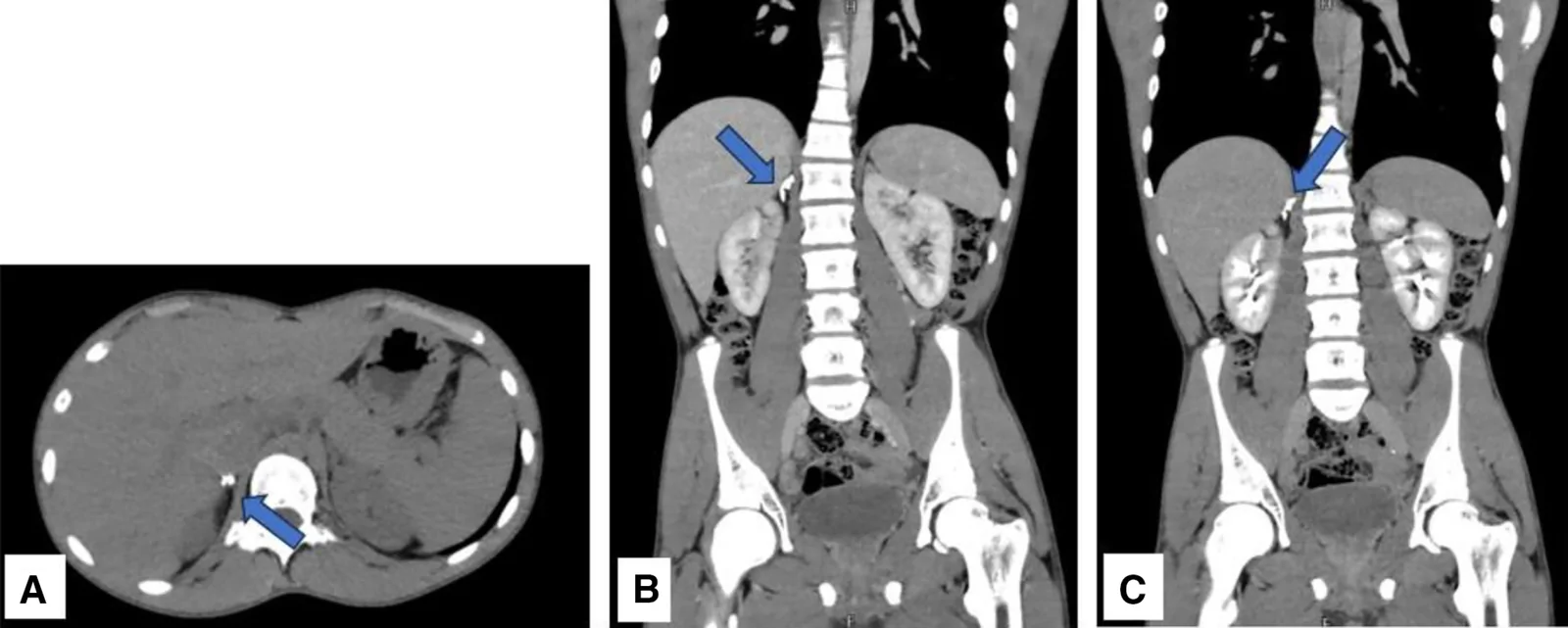
Follow-up CT scan obtained 9 months after adrenalectomy, displayed in (A) axial pre-contrast and (B, C) coronal venous and delayed phases, demonstrating surgical sutures at the operative bed of the removed right adrenal gland (blue arrow) with no evidence of local recurrence or new lesion. CT = computed tomography.

## 3. Discussion

This case illustrates the incidental presentation of AGN in an adolescent, discovered during trauma imaging rather than through endocrine symptoms. AGN is a rare, fully differentiated neuroblastic tumor that is usually identified only after surgical excision because preoperative distinction from other adrenal masses is difficult.^[4,5]^ Although AGN is more often reported in adults, adolescent cases warrant particular attention: adrenal masses are uncommon in young patients, the differential diagnosis includes less mature neuroblastic tumors, and current guidance supports a proactive approach to indeterminate adrenal lesions in this age group.^[8]^

The radiologic pattern in our patient was informative but should be interpreted in the context of the existing literature. On CT and MRI, the lesion showed progressive delayed enhancement without washout and no intracellular lipid. This “wash-in without wash-out” pattern, attributed to slow diffusion of contrast into the abundant myxoid and Schwannian stroma of ganglioneuroma, has been described previously in AGN, including in a dedicated report by Agrawal et al^[7]^ and in imaging series and reviews,^[6,9,10]^ and adolescent incidental cases have likewise been reported.^[10]^ Lipid-rich adenomas, by contrast, typically show low unenhanced attenuation, rapid washout, and signal loss on opposed-phase imaging.^[11,12]^ In our patient, the unenhanced attenuation of 20 HU, the absence of chemical-shift signal loss, and progressively increasing enhancement across the arterial, venous, and delayed phases argued against adenoma. We emphasize, however, that this pattern is suggestive rather than diagnostic: it is not specific to ganglioneuroma, overlap with pheochromocytoma and other adrenal neoplasms can occur,^[5,6]^ and a single case cannot validate any imaging feature as diagnostic. The present report should therefore be regarded as an additional imaging–pathology correlation in an adolescent rather than as establishing a novel imaging sign.

The differential diagnosis of an adrenal incidentaloma in an adolescent includes adenoma, pheochromocytoma, ACC, and the neuroblastic tumor spectrum. Pheochromocytoma was considered but was biochemically excluded, although imaging overlap may still occur in biochemically silent cases.^[11]^ ACC typically presents as a large, heterogeneous mass with necrosis, irregular margins, and possible local invasion; however, benign lesions such as AGN can occasionally mimic malignant features radiologically, and size alone cannot reliably exclude malignancy.^[13,14]^ Neuroblastoma and ganglioneuroblastoma remained relevant considerations given the patient’s age and could not be excluded preoperatively without tissue. In line with the European Society of Endocrinology recommendations, which advocate surgical resection of large, radiologically indeterminate adrenal masses and a proactive approach in young patients in whom adrenal incidentalomas are uncommon and the pretest probability of clinically relevant disease is higher, definitive resection was considered both diagnostic and therapeutic in this patient.^[8]^

Pathological examination remains the diagnostic gold standard. In the present case, the diagnosis rested primarily on morphology: a well-circumscribed tumor composed of mature ganglion cells within abundant Schwannian stroma, with no immature neuroblastic elements after extensive sampling – the defining features of ganglioneuroma within the neuroblastic tumor spectrum.^[1,15]^ S100 immunopositivity in both the Schwannian and ganglion cell components was supportive, consistent with the neural crest origin of the tumor, but was not by itself diagnostic; we have therefore framed the immunohistochemical findings as corroborative rather than confirmatory. The tan, whorled gross appearance and the histological predominance of fibrous and Schwannian stroma also correlate well with the progressive enhancement observed on imaging.^[9]^

A modest discrepancy was noted between the radiologic (approximately 6.1 cm × 3.6 cm × 5.0 cm) and gross pathologic (7.0 cm × 5.5 cm × 3.8 cm) measurements. Such differences are common and most likely reflect nonidentical measurement axes between imaging planes and the pathology bench, in vivo versus ex vivo conditions, and inclusion of residual adrenal and periadrenal tissue in the gross specimen. Importantly, both estimates place the lesion well above the size at which resection of a radiologically indeterminate adrenal mass is recommended, so the discrepancy did not affect the rationale for surgery.

Most AGNs are nonfunctioning, but hormonal evaluation is warranted whenever clinical or postoperative findings raise concern for endocrine activity.^[4,5,16]^ The mildly elevated preoperative plasma renin activity, in the setting of a normal aldosterone level and normal blood pressure, was considered nonspecific and may reflect posture, volume status after trauma, or assay variability. Postoperatively, a transient elevation of blood pressure was noted during follow-up; repeat 24-hour urinary metanephrines, normetanephrines, VMA, and catecholamines were normal, arguing against catecholamine excess from residual or occult chromaffin tissue. Because serial measurements were not systematically documented, the mechanism of this transient elevation remains undetermined, and we have avoided attributing it to the tumor or to its removal.

Complete surgical excision is the treatment of choice for AGN and, in published surgical series, is associated with an excellent prognosis and a very low recurrence risk.^[3,5,16]^ Our patient remains recurrence-free 9 months after surgery. We emphasize that this follow-up duration demonstrates a favorable short-term outcome but does not by itself establish long-term cure; the expectation of durable remission is based on the broader literature rather than on the present case, and longer follow-up would be required to confirm sustained remission in this patient.

This report has limitations beyond those inherent to a single case, and we state them explicitly. First, although multiphase CT documented progressively increasing attenuation from 20 HU (unenhanced) to 35 HU (arterial) and 55 HU (venous), with persistent further enhancement on 15-minute delayed images, the numeric delayed-phase attenuation value could not be retrieved from the archived imaging records; formal percentage-washout values therefore could not be calculated, and the absence of washout was assessed qualitatively. Second, the original laboratory reports, including assay methodology and institution-specific reference intervals, were not retrievable, so the biochemical results are reported as documented in the clinical record. Third, the transient postoperative blood pressure elevation was not captured with systematically documented serial measurements, and its mechanism remains undetermined. Finally, follow-up is limited to 9 months. Within these limits, the case contributes a documented adolescent example of AGN with correlated multiphase CT, adrenal-protocol MRI, gross and microscopic pathology, and postoperative imaging, and it illustrates a structured diagnostic approach to the uncommon problem of an adrenal incidentaloma in a young patient.

## 4. Conclusion

AGN is a rare, benign tumor that may present as an incidental adrenal mass, including in adolescents. Progressive delayed enhancement without washout and the absence of intracellular lipid on chemical-shift MRI – a pattern previously described in ganglioneuroma – may suggest a neurogenic origin, but imaging alone is insufficient for definitive diagnosis, and histopathological examination remains the gold standard. In our patient, complete surgical excision established the diagnosis and achieved a favorable short-term outcome, with no recurrence at 9 months; the excellent long-term prognosis and low recurrence risk of AGN are supported by the published literature rather than by this single case. Awareness of this entity can help clinicians refine the differential diagnosis of adrenal incidentalomas in young patients and guide appropriate management.

## Acknowledgments

The authors sincerely thank the patient and his family for their cooperation and for providing consent to share this case for educational and scientific purposes.

## Author contributions

**Conceptualization:** Muhammad Sweity, Izzeddin A Bakri.

**Data curation:** Wasef Alhroub, Mohammad Manasra, Ameer Qawasmi.

**Formal analysis:** Omair Bseiso.

**Methodology:** Omair Bseiso, Karam M Hmidat.

**Project administration:** Omair Bseiso, Sami Bannoura.

**Resources:** Omair Bseiso, Mohammad Alami, Abdallah Altell.

**Supervision:** Omair Bseiso, Aya Abedallah Ibrahim, Ammar Hassouneh, Sami Bannoura, Abdallah Altell.

**Validation:** Aya Abedallah Ibrahim, Hani Rjoob.

**Visualization:** Ammar Hassouneh.

**Writing – original draft:** Osaid Dabbas, Hani Rjoob, Ammar Hassouneh.

**Writing – review & editing:** Ameer Qawasmi, Ammar Hassouneh.
